# Percutaneous left atrial appendage occlusion in patients with a cardiac implantable electronic device

**DOI:** 10.1007/s10840-023-01512-0

**Published:** 2023-03-01

**Authors:** D. P. Staal, M. Maarse, E. Aarnink, M. F. M. Huijboom, B. G. S. Abeln, B. J. M. W. Rensing, M. J. Swaans, V. F. Van Dijk, L. V. A. Boersma

**Affiliations:** 1 Department of Cardiology, St. Antonius Hospital, Nieuwegein, the Netherlands; 2 Department of Cardiology, AMC Amsterdam, Amsterdam, the Netherlands

**Keywords:** Left atrial appendage occlusion, Cardiac implantable electronic device, Atrial fibrillation, Pacemaker, Implantable cardioverter defibrillator, Stroke

## Abstract

**Background:**

Left atrial appendage occlusion (LAAO) may be a viable option for stroke prevention in patients with non-valvular atrial fibrillation and a contraindication for oral anticoagulation. No evidence evaluating the safety of this procedure in patients with a cardiac implantable electronic device (CIED) exists. The aim of this study was to evaluate whether CIED function is affected by LAAO and to explore LAAO procedural characteristics and complications in patients with a CIED.

**Methods:**

This single-center cohort study included consecutive patients scheduled for percutaneous LAAO. Patients with a CIED prior to LAAO were selected and compared to the patients without CIED, concerning procedural characteristics and peri-procedural complications. In the group of patients with CIEDs, essential pacemaker integrity parameters were compared before and after the procedure to detect possible micro and macro lead displacements.

**Results:**

Thirty-one patients with CIED were scheduled for LAAO (age 73.7 ± 5.4 years, 65% males, CHA_2_DS_2_-VASc 4.3 ± 1.5, and HAS-BLED 3.3 ± 1.0). The 245 patients without CIED were younger, and HAS-BLED-score was slightly lower (69.4 ± 8.2 years, *p* < 0.001; 2.8 ± 1.0, *p* = 0.022). Patients without CIED more frequently underwent LAAO combined with catheter ablation (*p* = 0.002). All other procedural characteristics were comparable between both groups. No visible lead displacement was observed on chest X-ray after LAAO. Additionally, no differences in impedance, threshold, or intracardiac sensing in various CIED lead locations were found prior versus post LAAO.

**Conclusion:**

This study supports the feasibility and safety of LAAO in patients with a CIED.

## Introduction

Current international guidelines recommend the use of oral anticoagulation (OAC) to prevent thrombo-embolic complications in patients with AF [1] and an increased thrombo-embolic risk based on the CHA_2_DS_2_-VASc score [2–5]. Mechanical closure of the left atrial appendage (LAA), considered the main source of thrombi, with a percutaneous closure device is a rising alternative for patients with a contraindication for long-term OAC [6]. Several randomized controlled trials and registries demonstrated non-inferiority of left atrial appendage occlusion (LAAO) compared to vitamin K antagonists for stroke prevention in patients with non-valvular AF [7–9].

The population treated with LAAO is diverse and patients frequently suffer from several comorbidities. The need for LAAO in patients with a cardiac implantable electronic device (CIED) with transvenous leads (TVL), such as a pacemaker or implanted cardioverter defibrillator (ICD), is therefore not uncommon. During the percutaneous LAAO procedure, the closure device is led up to the right atrium through the femoral vein, followed by puncturing of the atrial septum to access the LA and approach the LAA [10]. The presence of intracardiac pacing or defibrillation leads in patients with a CIED, especially in the right atrium, may complicate the LAAO procedure. The leads possibly limit manoeuvrability of the transseptal sheath and the LAAO device delivery system. Difficult transseptal puncture (TSP) increases the risk of procedural complications such as tamponade. Additionally, the different percutaneous catheters used during the LAAO procedure may manipulate the present TVL and therewith influence CIED performance.

There is few published data on the safety of LAAO in patients with a CIED. The purpose of this study was to evaluate whether CIED performance is affected by LAAO and to explore whether the presence of a CIED causes LAAO procedural safety concerns.

## Methods

### Study design

This prospective single-center cohort study included consecutive patients scheduled for percutaneous LAAO between 2009 and 2021 in the St. Antonius hospital, the Netherlands. Patients with a CIED with TVL implanted prior to LAAO were selected and compared to all other patients (without CIED) scheduled for LAAO. Stand-alone LAAO procedures and LAAO combined with catheter ablation for AF were included. Data was collected using a web-based database containing patient demographics, medical history, LAAO procedural characteristics, peri-procedural complications, peri-procedural chest X-rays, and measurements on essential CIED integrity parameters before and after LAAO. The study was conducted according to the Declaration of Helsinki and approved by the local ethics committee (MEC-U).

### Patient and procedural management

Eligibility for percutaneous LAAO was assessed by the cardio-electrophysiologist. All patients underwent transesophageal echocardiography (TOE) to rule out intracardiac thrombus, evaluate LAA anatomy, and guide the LAAO device implantation. LAAO device type was determined at the discretion of the implanting physician, either WATCHMAN 2.5 (Boston Scientific, Natick, MA, USA), WATCHMAN FLX (Boston Scientific, Natick, MA, USA), or Amplatzer Amulet (Abbott, Minneapolis, MN, USA). The LAAO procedure has been described in detail in previous literature [11]. For patients who underwent a combined LAAO with catheter ablation, catheter ablation was performed prior to LAAO. All patients underwent chest X-ray after LAAO to confirm intracardiac positioning of the LAAO device and rule out TVL displacements before discharge. Procedural success rate was verified by follow-up TOE, or cardiac computed tomography (CT) was performed between 45 days and six months after LAAO.

### Outcomes

The main goal was to evaluate safety and feasibility of LAAO in patients with a CIED and to identify differences in peri-procedural complications with patients without a CIED. The efficacy outcome was described as acute procedural success of LAAO, defined as adequate LAA closure according to the manufacturer’s instructions for use; device deployed and implanted in correct position, meeting all release criteria and with no significant peri-device leakage (≤ 5 mm for WATCHMAN devices and ≤ 3 mm for Amplatzer Amulet devices).

The primary safety outcome was the occurrence of peri-procedural complications of LAAO. Peri-procedural complications were defined as any deviation of standard procedure or complication occurring within 7 days after LAAO procedure. The secondary safety outcome was the occurrence of CIED macro or micro lead displacement. Macro lead displacement encompasses pacing failure or untreated arrhythmias in the post-procedural period [12], and was detected visually by comparing lead position on chest X-ray prior to LAAO and after LAAO [13]. To detect possible micro lead displacements, measurements of impedance, threshold, and intracardiac sensing of the different TVL of the CIEDs before and after LAAO were compared [14]. Furthermore, time from venous puncture until TSP, time from venous puncture until sheath removal, fluoroscopy times, and radiation exposure (DAP in Gy·cm^2^) were measured to evaluate if the presence of TVL prolongs procedure times.

### Statistical analysis

Baseline and procedural characteristics are presented as mean ± standard deviation (SD) or median with interquartile range (IQR) for continuous variables. Differences between variables are examined using Student’s *t-*test or Mann–Whitney *U* as appropriate. Categorical variables are presented as numbers with percentages and were compared using the *χ*^2^ test. The Fisher exact test was used when the expected count was less than 5 in > 20% of all cells. Paired comparisons were analyzed using a paired *t*-test for normally distributed variables. A *p* value of 0.05 or less was considered statistically significant. Statistical analyses were conducted using SPSS version 26.0 (Statistical Package for Social Sciences, Chicago, IL, USA).

## Results

### Baseline characteristics

**Fig. 1:**
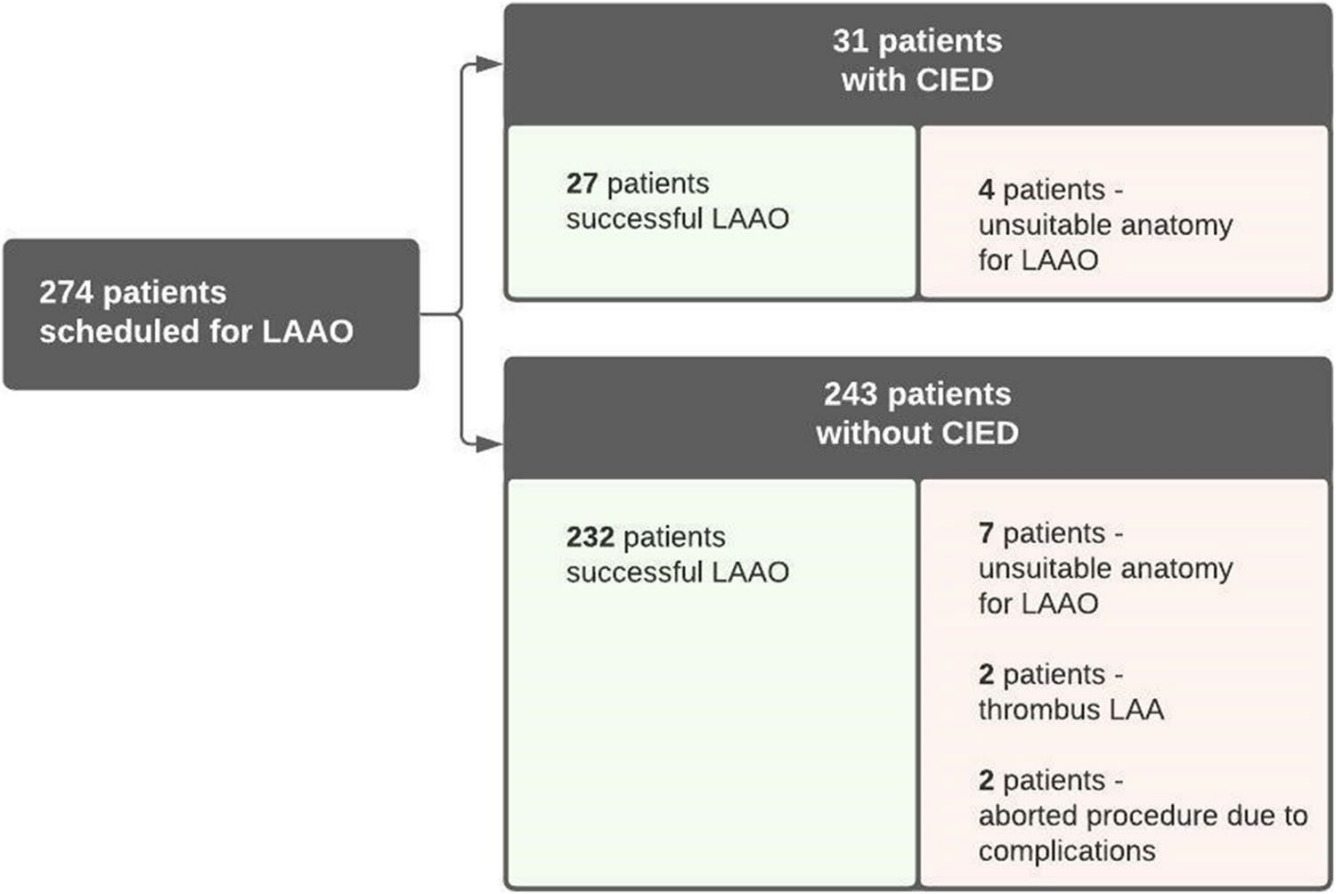
Flowchart patient inclusion. CIED: cardiac implantable electronic device; LAAO: left atrial appendage occlusion

A total of 276 consecutive patients underwent LAAO, of whom 31 (11%) with a CIED in situ prior to the LAAO procedure (flowchart Fig. 1). Twenty-seven out of 31 (87%) patients had pacemaker leads, 24 of 31 patients had leads in the right atrium, and 7 of 31 patients had defibrillator leads. Specification of the device settings before LAAO can be found in Table 1. Patients with and without CIED were similar regarding gender (*p* = 0.831) and type of AF (*p* = 0.736). In both groups, participants were at high risk for thrombotic events (mean CHA_2_DS_2_–VASc 4.3 vs. 3.8, *p* = 0.104). Patients with CIED were slightly older (mean age 73.7 ± 5.4 vs. 69.4 ± 8.2, *p* < 0.001), and bleeding risk was slightly higher (mean HAS-BLED 3.3 ± 1.0 vs. 2.8 ± 1.0, *p* = 0.022). All baseline characteristics can be found in Table 2.

**Table 1:** CIED specification

	Group I with CIED (%)
VVI	3 (10.3)
DDD	19 (65.5)
CRT-D	2 (6.9)
CRT-D (with epicardial LV-lead)	3 (10.3)
ICD	2 (6.9)

*CIED*, cardiac implantable electronic device; *CRT-D*, cardiac resynchronization therapy defibrillator; *DDD*, dual-chamber antibradycardia; *ICD*, implantable cardioverter-defibrillator; *LV*, left ventricle

**Table 2:** Baseline characteristics

	Group I with CIED (%)	Group II without CIED (%)	*p* value
Total number of patients	31	243	
Gender male	20 (64.5)	152 (62.6)	0.831
Age in years	73.7 ± 5.4	69.3 ± 8.2	< 0.001
AF-type			
Paroxysmal AF	16 (51.6)	118 (48.6)	0.736
Persistent	5 (16.1)	54 (22.2)	
Permanent	10 (32.3)	71 (29.2)	
CHA_2_DS_2_-VASc (mean ± SD)	4.3 ± 1.5	3.8 ± 1.5	0.104
Low–intermediate risk (0–3)	9 (29.0)	111 (45.7)	0.079
High risk (> 3)	22 (71.0)	132 (54.3)	
HAS-BLED	3.3 ± 1.0	2.8 ± 1.0	0.021
Low–intermediate risk (0–3)	17 (54.8)	188 (77.4)	0.007
High risk (> 3)	14 (45.2)	55 (22.6)	
History of ischemic stroke	7 (22.6)	80 (32.9)	0.244
History of major bleeding:	26 (83.9)	134 (55.1)	0.002
Intracranial haemorrhage	15 (48.4)	75 (30.9)	0.050
Other	13 (41.9)	66 (27.2)	0.087
APT or (N)OAC use prior to LAAO ^*^:			
None	5 (16.1)	42 (17.3)	0.872
Aspirin	13 (44.8)	46 (20.5)	0.004
Clopidogrel	3 (9.7)	28 (11.5)	1.000
VKA	7 (22.6)	98 (40.3)	0.056
NOAC	4 (12.9)	41 (16.9)	0.574
Persantin	2 (6.6)	4 (1.6)	0.139

*AF*, atrial fibrillation; *CIED*, cardiac implantable electronic device; *(N)OAC*, (novel) oral anticoagulation; *SD*, standard deviation; *VKA*, vitamin K antagonist

^*^Medication use scored at day directly before LAAO

### Left atrial appendage occlusion: procedural outcomes

The procedural success rate of LAAO was 27/31 among CIED patients compared to 232/243 in the control group (87% versus 96%, *p* = 0.075). All four failed procedures in group I were unsuccessful due to unsuitable anatomy for adequate LAAO meeting all release criteria. The first procedure was terminated since the device could not be anchored in the desired position due to the large size of the LAA. During the second procedure, the diameter of the LAA was very small and the required access route was not obtainable due to angulation of the heart. The third patients’ LAA consisted of a wide base without limbus with an acute turn to a narrow point; no proper landing zone for the device was present. The fourth LAAO procedure failed because of insufficient depth, due to a very proximal bifurcation, which limited deployment of the closure device. The presence of TVL of a CIED was not indicated as a problem by the operating physician in any of the LAAO procedures. The results show no statistical difference in complete closure between the CIED patients (*n* = 21) compared to patients without CIED (n = 185), versus minimal residual flow (*n* = 4; *n* = 29, *p* value = 0.754). All procedural characteristics presented in Table 3 were comparable between groups, except that patients with a CIED underwent stand-alone LAAO more frequently compared to patients without CIED (74% vs. 45%, *p* = 0.002). Peri-procedural complications were equally observed in both groups (13.8 vs. 14.7%; *p* value 1.000). The operating physician did not indicate any complication occurring in patients with a CIED to be related to the presence of TVL. Specifications of all complications observed are described in Table 3.

**Table 3:** LAAO procedural characteristics and outcomes

	Group I with CIED (%)	Group II without CIED (%)	*p* value
Total number of patients	31	243	
Procedural success rate	27 (87.1)	232 (95.5)	0.075
Procedure type			
Stand-alone LAAO	23 (74.2)	110 (45.3)	0.002
Combined CA with LAAO	8 (25.8)	133 (54.7)	
Device type			
WATCHMAN 2.5	25 (80.6)	189 (77.8)	0.792
WATCHMAN FLX	4 (12.9)	42 (17.3)	
Amplatzer Amulet	2 (6.5)	12 (4.9)	
Duration procedure in minutes, mean (SD)			
Stand-alone LAAO			
Venous puncture–TSP	10: 40 ± 05: 23	12: 56 ± 10: 16	0.319
Total procedure time	53: 52 ± 20: 50	53: 27 ± 21: 48	0.934
Fluoroscopy time	09: 59 ± 05: 53	08: 48 ± 04: 54	0.358
Combined CA with LAAO			
Venous puncture–TSP	08: 00 ± 03: 52	09: 35 ± 07: 35	0.583
Total procedure time	1: 45: 00 ± 29: 11	1: 47: 31 ± 30: 11	0.818
Fluoroscopy time	13: 08 ± 04: 50	15: 37 ± 05: 56	0.281
Radiation exposure (DAP Gy·cm^2^)			
Stand-alone LAAO	21.5 (12.3–36.9)	20.0 (11.0–33.0)	0.381
Combined CA with LAAO	29.0 (18.0–38.0)	37.0 (25.0–57.3)	0.157

*CA*, catheter ablation; *CIED*, cardiac implantable electronic device; *DAP*, dose area product; *LA*, left atrium; *LAA*, left atrial appendage; *LAAO*, left atrial appendage occlusion; *NSVT*, non-sustained ventricular tachycardia; *SD*, standard deviation; *TIA*, transient ischemic attack; *TOE*, transesophageal echography; *TSP*, transseptal puncture

### CIED measurements: macro and micro lead displacement

**Fig. 2:**
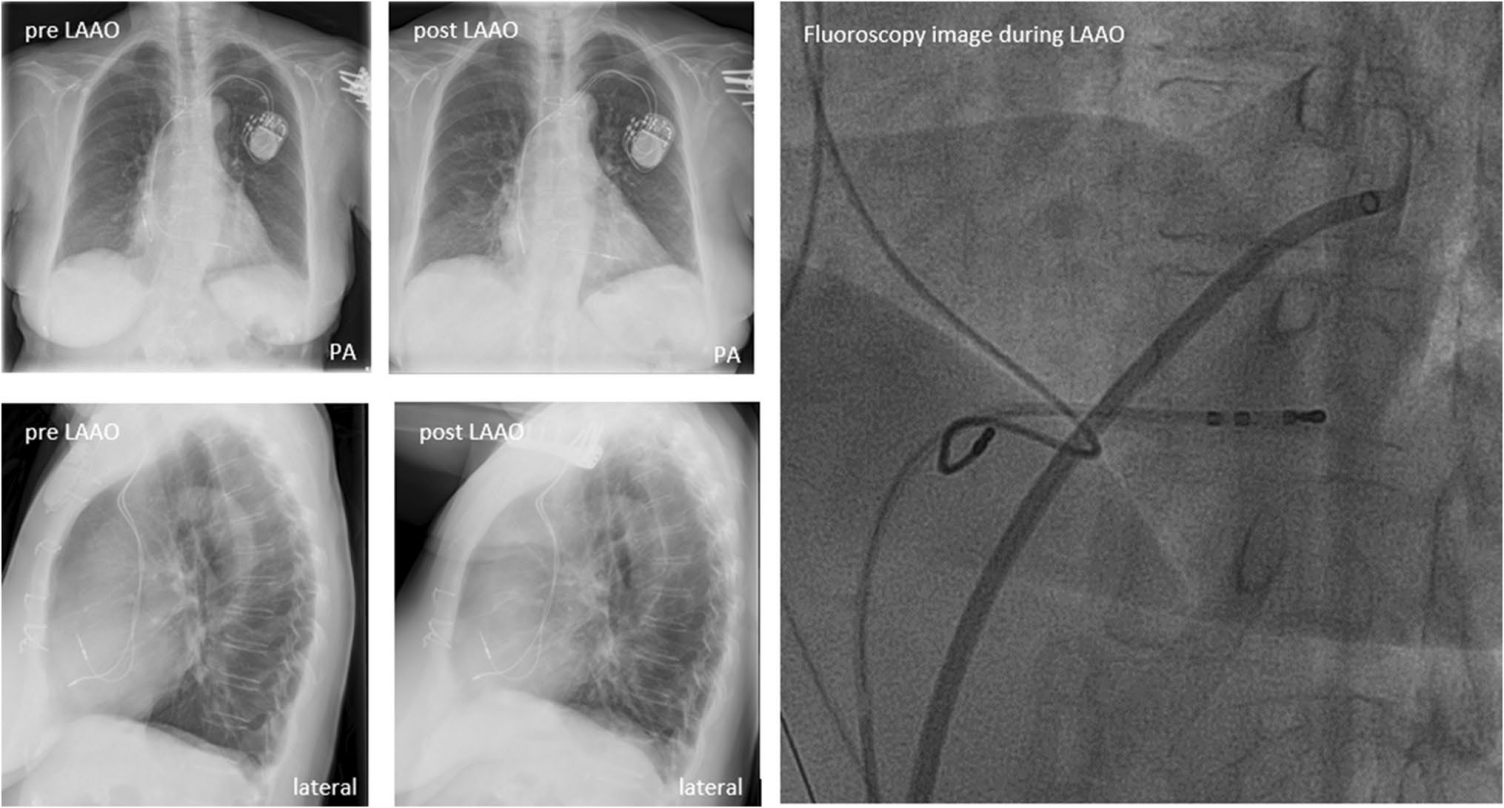
Typical examples patient with CIED and TVL. (Left) Chest X-rays posterior–anterior view and lateral view, prior, and post LAAO with CIED and TVL in situ. (Right) Fluoroscopy imaging showing TVL and access sheath percutaneous LAAO. CIED: cardiac implantable electronic device; LAAO: left atrial appendage occlusion; PA: posterior–anterior; TVL: transvenous leads

No macro lead displacements were identified; no visual displacements of the CIED leads were observed at chest X-ray (typical example Fig. 2) and no pacing failure or arrhythmia treatment failure was detected in the post-procedural period. In one patient with a VVI pacemaker that underwent radiofrequency catheter ablation combined with LAAO, the rate-responsiveness mode of the pacemaker was switched off before but not restored after LAAO and the patient developed complaints of fatigue during exercise after LAAO. During the next CIED visit, this problem was detected and solved. Overall, no statistical difference of impedance, threshold, and intracardiac sensing was observed between pre- and post-LAAO measurements, suggesting that no micro lead displacement occurred due to LAAO (Tables 4 and 5). The median duration of lead implantation was 32 months (IQR: 10–57 months) between CIED implantation and LAAO, with a minimum of 2 weeks. The median time of the last pacemaker follow-up before LAAO procedure was 78 days (IQR: 38–145) and 62 days (IQR: 7–125 days) between the first pacemaker follow-up after LAAO procedure.

**Table 4:** Procedural complications

	Group I with CIED (%)	Group II without CIED (%)	*p* value
Total number of patients	31 (11.3)	243 (88.7)	
Total number of procedures with any complication:	4 (12.9)	33 (13.5)	1.000
Procedural complications:			
Vascular access-related complications	2 (6.5)	14 (5.8)	
Peri-cardial effusion/tamponade	0 (0.0)	5 (2.1)	
Air embolism	0 (0.0)	4 (1.6)	
Device embolization	0 (0.0)	2 (0.8)	
Thrombus (LA/LAA/sheath)	1 (3.2)	2 (0.8)	
TIA/ischemic stroke	1 (3.2)	2 (0.8)	
TOE probe complication	1 (3.2)	1 (0.4)	
Sensoring CIED post-LAAO not activated	1 (3.2)	0 (0.0)	
NSVT detected during hospitalization	0 (0.0)	1 (0.4)	
New left bundle branch block	0 (0.0)	2 (0.8)	
Phlebitis	0 (0.0)	1 (0.4)	
Fever/elevated CRP unknown cause	0 (0.0)	1 (0.4)	
Gastro-intestinal bleeding (BARC 3b)	0 (0.0)	1 (0.4)	

*BARC*, bleeding academic research consortium; *CIED*, cardiac implantable electronic device; *CPR*, C-reactive protein; *LA*, left atrium; *LAA*, left atrial appendage; *LAAO*, left atrial appendage occlusion; *TIA*, transient ischemic attack; *TOE*, transesophageal echography; *TSP*, transseptal puncture

**Table 5:** CIED lead function measurements

	*n*	Pre-LAAO	Post-LAAO	Degree of change	
	Total *n*	Mean ± SD	Mean ± SD	Mean ± SD	*p* value
Right atrium					
Impedance (Ω)	19/22	461 ± 110	457 ± 106	− 4.4 ± 28	0.507
Threshold (V)	14/22	0.85 ± 0.45	0.89 ± 0.52	0.05 ± 0.21	0.430
Sensing (mV)	15/22	2.42 ± 1.47	3.15 ± 1.70	0.73 ± 1.43	0.069
Right ventricle					
Impedance (Ω)	26/31	528 ± 165	550 ± 215	21 ± 122	0.374
Threshold (V)	26/31	1.08 ± 0.75	1.11 ± 0.72	0.03 ± 0.38	0.674
Sensing (mV)	22/31	9.86 ± 4.56	10.47 ± 5.84	0.60 ± 4.98	0.576
Left ventricle					
Impedance (Ω)	4/5	472 ± 247	452 ± 221	− 20 ± 42	0.417
Threshold (V)	3/5	1.42 ± 0.45	1.37 ± 0.32	− 0.05 ± 0.18	0.678
Sensing (mV)	2/5	21.90 ± 2.69	19.8 ± 0.28	2.10 ± 2.40	0.433

*CIED*, cardiac implantable electronic device; *LAAO*, left atrial appendage occlusion; *SD*, standard deviation

## Discussion

This observational study is the first registry reporting on feasibility and safety of LAAO in patients with CIEDs and TVL. Although the main outcomes showed a trend towards more procedural LAAO failures in the patients with CIED, this was due to unsuitable anatomy for LAAO rather than obstruction by the CIED. Procedural complications, duration of procedure, and radiation exposure did not significantly differ between patients with and without CIED. Neither macro, nor micro lead displacement occurred during LAAO in patients with CIED, indicating that CIED performance was unaffected by LAAO. Overall, our study does not raise additional safety concerns for LAAO in patients with a previously implanted CIED.

The procedural success rates in both groups were somewhat lower in comparison to other large LAAO registries such as NCDR LAAO (98.3%) and EWOLUTION (98.5%) [7, 15]. The lack of pre-procedural imaging for assessing suitable anatomy may partially explain the difference in success rates, as this is not routinely performed in our center. Glikson et al. described that 25% of the patients were rejected prior to the procedure by pre-procedural imaging, due to exclusion criteria of LAA anatomy (orientation, size, shape, and width of the LAA) [10]. The aborted procedures in both groups might have been avoided, if patients with unsuitable anatomy were excluded prior to LAAO by preprocedural imaging.

In the present study, we found no difference in procedural complications between patients with and without CIED (13.8% vs 14.7%, *p* value: 1.000). Our complication rate is higher than the NCDR LAAO registry, which reported 2.8% in-hospital major adverse events [15]. However, these results are difficult to compare as the definition of complications is different. In our study, we defined procedural complications as any deviation of standard procedure, while the NCDR only recorded major adverse in-hospital events. Nonetheless, one single complication stood out and was specific for patients with a CIED: the programming of the CIED was not restored a procedure which included ablation. During CA procedure, the CIED is deactivated as a precautionary measure to prevent oversensing and inadequate pacing or defibrillation shocks [16]. Therefore, this incident is no risk for LAAO specifically, but only for procedures combined with catheter ablation.

Previous literature suggests precautionary measures for CA procedures in patients with CIED, which could be adopted for LAAO in patients with CIED [16]. First of all, these studies suggest that the CA procedure should be delayed until the TVL have obtained a stable healing and positon. A minimum of 6 weeks and ideally 3 months are recommended for TVL maturation [16–18]. Secondly, to avoid TVL damage and displacement extra caution should be taken while manipulating the transseptal sheath during TSP.

In a study among 86 AF patients with CIED undergoing CA, Lakkireddy et al. reported no statistical difference in procedural complications in CIED in comparison to a control group undergoing CA for AF [17]. This observation is in line with our findings, showing no statistically significant difference in procedural complications between patients with or without CIED who underwent LAAO (13.8% vs. 14.7%, *p* value = 1.000). Moreover, Lakkireddy et al. described complications such as pulmonary vein stenosis (2%), pulmonary edema (1%), and stroke (1%) in patients with CIED [17]. In our study, no pulmonary vein stenosis cases and pulmonary oedema were observed, since these CA-related complications are rare and only detectable with CT [18]. Likewise, in our study, there was no indication of different TIA/stroke occurrence in patients with CIED (*n* = 1) and patients without CIED (*n* = 2).

In theory, LAAO may be more complex in patients with CIED and TVL, although this is not confirmed by our study’s results. The procedure’s duration, fluoroscopy time, and radiation exposure were comparable between patients with and without CIED in both stand-alone LAAO and combined LAAO and CA procedures (Table 3). This is in line with the findings of Lakireddy et al., who also observed no statistical difference in procedure time between patients with and without CIED [17].

Lakkireddy et al. reported two atrial lead dislodgements both were attributed to recent lead introduction [17]. More recently, Dinshaw et al. studied 190 patients with CIED undergoing CA for AF and showed 4.7% lead dislodgement, 1.1% lead fracture, and 1.1% lead insulation during long-term AF follow-up after CA using continuous atrial rhythm monitoring [19]. In our study, no macro or micro lead displacement occurred following LAAO in patients with CIED. Although a numerical difference was observed between pre-LAAO (2.42 ± 1.47) and post-LAAO sensing in the RA (3.15 ± 1.70), this trend was not significant (*p* value: 0.069). This could be explained by the limited sample size of CIED patients, operator skill, or chance. Another explanatory factor could be procedural differences between LAAO and CA. For example, during CA electromagnetic interference could occur, which was suggested to possibly result in micro lead displacement [20]. This would be unlikely to occur during LAAO since no radio frequent current or other form of CA is used for LAAO, besides the mechanical force.

### Limitations

Several limitations should be considered for interpreting the results of this study. Despite the prospective study design, CIED performance parameters were gathered retrospectively, resulting in some missing data. The limited sample size of the cohort of patients with CIED, missing data, and rare occurrence of procedural complications during LAAO undermine the power of the analysis. Additionally, since the effects of LAAO on CIED macro and micro lead displacement were not assessed at pre-defined time intervals directly before and after LAAO, other factors may have influenced pacemaker integrity parameters. Likewise, later originating subtle CIED parameters disruptions may have been missed due to the limited follow-up time.

## Conclusion

This study supports the safety and feasibility of LAAO in patients with CIED. Future studies evaluating procedural outcomes and complications and macro/micro lead displacements in a larger sample are needed to further validate our findings.

## Data Availability

The data that support the findings of this study are available from the corresponding author, D.P. Staal, upon reasonable request.
